# Burden of illness and quality of life in patients being treated for seasonal allergic rhinitis: a cohort survey

**DOI:** 10.1186/2045-7022-3-33

**Published:** 2013-10-09

**Authors:** Mark Small, James Piercy, Pascal Demoly, Helen Marsden

**Affiliations:** 1 Adelphi Real World, Macclesfield, UK; 2 University Hospital of Montpellier, Inserm U657, France; 3 HM – GlaxoSmithKline, Stockley Park, Uxbridge, UK

**Keywords:** Seasonal allergic rhinitis, Symptom-free days, Intra-nasal steroids

## Abstract

**Background:**

Allergic Rhinitis is an inflammatory disease which is characterised by burdensome nasal and/or ocular symptoms. This study aimed to assess the impact of symptoms (number of symptom-free days (SFD) and Quality of Life (QoL)) in patients with Seasonal Allergic Rhinitis (SAR) being treated with fluticasone furoate (FF), mometasone furoate (MF) or fluticasone propionate (FP).

**Methods:**

In a cross-sectional, non-interventional, cohort analysis, primary care physicians and allergy specialists in France, Germany, and Spain were recruited via telephone interviews. Each physician prospectively recruited 4 SAR patients - 2 receiving FF, 1 receiving MF and 1 receiving FP - during June 2009. Patients answered questions on symptoms and completed questionnaires on QoL (mini-rhinoconjunctivitis Quality of Life Questionnaire, RQLQ) and burden of illness (Pittsburgh Sleep Quality Index).

**Results:**

A total of 540 patients were recruited during June 2009. 88 patients were subsequently found to be ineligible and excluded from the analyses. In the 4 weeks prior to assessment, patients reported a mean of 14.58 (±8.42) SFD. Patients receiving FF had more SFD (mean 15.45 ±8.29) than patients receiving MF (adjusted mean difference -1.22, 95% Confidence Interval (CI) [-3.16 to 0.72], p=0.434) or FP (adjusted mean difference -1.95, 95% CI [-3.87 to -0.03], p=0.092), although statistical significance was not achieved. The mean RQLQ score was 1.54 (±1.06). Patients receiving FF had a better quality of life in the previous week (mini-RQLQ score: mean 1.42, ±1.04) than patients receiving MF (adjusted mean difference 0.28, 95% CI [0.03 to 0.52], p=0.052) or FP (adjusted mean difference 0.18, 95% CI [-0.05 to 0.41], p=0.244). Again, none of these results achieved statistical significance.

**Conclusions:**

At the height of the allergy season, patients with SAR suffer symptoms approximately 50% of the time, and report an impact on their QoL. No significant differences were observed between FF, FP and MF related to SFD or QoL.

**Trial registration:**

ClinicalTrials.gov identifier: NCT01199757

## Background

Allergic rhinitis (AR) is a common inflammatory condition of the upper respiratory tract, nasal cavity and eyes affecting up to 20% of the population in the United States (US) and Europe [1]. AR is characterized by both nasal and ocular symptoms including rhinorrhoea, sneezing, itchy/blocked nose, sinus pressure, itchy/red eyes, snoring and other sleep problems. Across Europe up to 71% of AR patients suffer from both nasal and ocular symptoms [2], especially (but not only) those having seasonal AR (SAR) and one in five of these (21%) indicated that this was their most bothersome symptom [2].

The bothersome nature of AR symptoms can severely affect daily activities including ability to work [3], examination performance [4,5], impact on Quality of Life (QoL) and psychosocial well being [6,7]. Patients report that ocular symptoms are both troublesome [8] and often inadequately controlled [2] and it has been demonstrated that the added presence of ocular symptoms in AR patients suffering with nasal symptoms deteriorates patients’ QoL, leads to greater loss of productivity and places higher burden on resource utilization [9]. SAR patients suffer predominantly during the pollen season, limiting the window during which the impact of the disease and the effect of treatment can be assessed.

Intranasal corticosteroids (INS) have been shown to decrease ocular symptoms associated with AR as well as nasal symptoms [10-12]. Similarly, non-sedating antihistamines have been shown to be effective at controlling ocular symptoms of AR patients [13]. Comparative research indicates that INSs provide equal or greater relief of ocular allergy symptoms compared with intranasal or oral antihistamines [14]. However, antihistamines generally have their greatest efficacy against early-phase, histamine-mediated symptoms (e.g. itching, rhinorrhoea) and lesser efficacy in treating late phase symptoms (e.g. congestion) [11]. Recent patient preference studies have shown that patients have high expectations of their anti-allergic treatment, desiring attributes such as good symptom relief, quick-onset and long-lasting effects and favourable side effect profile [15,16]. However, patients are often dissatisfied with the efficacy of their treatment which can lead to poor compliance and supplementation with over the counter products [16]. A study of patients under specialist care reported that patients preferred nasal spray to oral treatment, however feared adverse events (such as habituation, damage to mucous membranes, addiction and influence on other organs) of INS therapies [15]. This data highlights the need for INS treatments to have good all round efficacy, with a reassuring safety profile, to provide a holistic treatment for AR that improve the patients’ quality of life.

Several studies of INS treatments have looked at their impact on quality of life [17-21]. INS therapies have generally been reported to have a clinically meaningful improvement in QoL, as measured by the Rhinoconjunctivitis Quality of Life Questionnaire, compared to placebo. However few studies have compared the impact of different INS therapies on symptoms and quality of life.

Here we report the results of a real world survey of treated SAR patients focusing on the occurrence of Symptom-free days (SFD) over the previous 4 weeks and quality of life over the previous week, and comparing three commonly used INS treatments. The study also looked at the impact of INS therapies on: work productivity, sleep, visits to health care professionals and out of pocket expenditure on over the counter medications.

## Methods

This was a cross-sectional, non-interventional, cohort survey analysis, conducted in June 2009 conducted by Adelphi Real World. ClinicalTrials.gov identifier: NCT01199757.

A random sample of 30 Primary Care Physicians (PCPs) and 15 allergy specialists in France, Germany, and Spain were recruited via telephone interviews by local agencies. To maximise generalisability, our physician recruitment criteria were as broad as possible so to not limit our primary care physician population to those who specialised in allergy. The only inclusion criteria were that each physician had to have qualified after 1970, see three or more AR patients per week, be personally responsible for treatment decisions for their AR patients and agree to participate.

Each physician prospectively recruited four consecutive consulting SAR patients over the age of 12 who were receiving, and had received at least one full prescription of, specified INS therapy and who agreed to participate in the research. Patients with a co-diagnosis of asthma or Chronic Obstructive Pulmonary Disease (COPD) were excluded. Two patients receiving FF [Avamys™], one patient receiving mometasone furoate (MF) [Nasonex™] and one patient receiving fluticasone propionate (FP) [Flixotide™] were recruited (in any order) by each physician. The survey was conducted as research in accordance with and defined by the European Pharmaceutical Market Research Association (EphMRA) code of conduct for international healthcare market research, so ethical approvals were not required. In line with EphMRA requirements, all participating patients provided informed consent. No tests or investigations were performed as part of this research, and to ensure compliance with data protection laws, all data were de-identified and aggregated prior to receipt by Adelphi Real World.

Physicians recorded data relating to patient characteristics including demographics, disease classification as defined by the ARIA (Allergic Rhinitis and its Impact on Asthma) guidelines [22], symptoms, patient visits, INS treatment (including whether for ocular and/or nasal symptoms) and co-prescription. Physicians were asked to capture this information by ticking a pre-coded list. The provided information was based on the assessment by the physician whether this be from the most recent consultation or with recourse to patient notes.

Each patient was asked to complete a matched patient-completion forms independent from the physician on symptoms over the previous 4 weeks, and QoL and burden of illness questionnaires: mini-rhinoconjunctivitis Quality of Life Questionnaire (RQLQ), Pittsburgh Sleep Quality Index (PSQI), Work Productivity and Activity Impairment: Allergy Specific questionnaire plus Classroom Impairment Questions: Allergy Specific (WPAI+CIQ:AS). The number of work days lost, PCP or specialist visits due to AR, and over-the-counter (OTC) medicines used over the previous 4 weeks were also recorded.

The mini-RQLQ [23] assesses QoL over the previous week. It is comprised of 14 items, in five domains (Activity Limitations, Practical Problems, Nose Symptoms, Eye Symptoms and Other Symptoms), each evaluated on a seven point scale (0 = “Not troubled”, 6 =“Extremely troubled”). The overall QoL score is the mean score across all 14 items and the Minimal Important Difference is 0.7.

The PSQI [24] assesses sleep quality and disturbance over the previous month. It is split into seven components: Subjective sleep quality; Sleep latency; Sleep duration; Habitual sleep efficiency; Sleep disturbance; Use of sleep medication; Daytime dysfunction. Each component score ranges from 0 (no difficulty) to 3 (severe difficulty). The final PSQI global score is derived from summing these seven component scores (range 0 to 21). A score of >5 is suggestive of significant sleep disturbance, but no minimally important difference has been reported.

The WPAI+CIQ:AS [25] assesses work, classroom and activity impairment over the previous seven days. This has nine questions which form three domains (work for working people, study for students and activity impairment for both subgroups). Seven separate ‘scores’ are produced which related to: Percent work time missed due to allergy; Percent impairment while working due to allergy; Percent overall work impairment due to allergy; Percent class time missed due to allergy; Percent impairment in the classroom due to allergy; Percent regular activity (other than work or classes) impairment due to allergy.

Each patient was asked how much they had spent (in Euros) on medication for their AR from a pharmacy or supermarket that had not been prescribed by their doctor between over the previous 3 months.

The sample size was chosen based on the practicalities of conducting the study, and was designed to ensure a spread of patients across both primary care and specialist care, and between the therapies of interest. No formal sample size or power calculations were made. The 2:1:1 ratio was chosen to collect a higher number of patients on FF as it was planned to compare FF against MP and FP both separately and combined. Please note that only patients who were already receiving these medications were included, therefore there was no possibility for physicians to initiate treatment of any kind in order to include patients in the study.

Ordinary Least Square (OLS) regression models were used on the following outcome variables: number of SFD over the past four weeks, mini-RQLQ, PSQI, WPAI+CIQ:AS, number of work days lost, number of healthcare professional (HCP) consultations (physician reported), number of HCP consultations (patient reported), number of AR treatments, number of OTC products used and total OTC spend. Variables such as age, gender, Body Mass Index (BMI), number of concomitant conditions since March 2009, number of AR drugs currently prescribed and ARIA severity were included in the model to control for any influence they may have had on the results of generic assessments such as time off work, and overall health status.

All analyses were conducted on the total study population, and by country, ARIA severity Bonferroni corrections were made for the multiple comparisons but no adjustments were made for missing data; results presented are based on available data only. All statistical analyses were conducted in Stata Version 10.1.

A post-hoc analysis on a sub-group of patients who had experienced both ocular and nasal symptoms was conducted. Nasal and ocular patients were defined as those who had ever suffered with itchy/red eyes and/or watery eyes in addition to having nasal symptoms (as defined by the symptoms provided by the physician).

## Results

### Population

540 SAR patients were recruited into the study. Subsequently 88 patients were found to be on more than one INS, and were excluded from the analyses. The study population, where demographic information was available, was 52.2% female, had a mean age of 36.2 (±13.9) years, and had a mean BMI of 24.1 (±3.7), which was comparable across the INS groups (Table 1). Our study population was made up of mostly (54.5%) moderate/severe persistent patients, with only a few (12.2%) mild intermittent patients. The mean number of co-morbidities reported during the study period was 0.96 (±1.14), and the mean number of concomitant medications taken in the same period was 1.60 (±0.67) - both of which were similar across the treatment groups.

**Table 1:** Patient population characteristics

	**Total**	**FF**	**MF**	**FP**
	**N=452**	**N=229**	**N=108**	**N=115**
Age (Mean, (SD))	36.2 (13.9)	36.5 (13.7)	35.8 (13.7)	36.1 (14.6)
Gender (% females)	52.2%	49.8%	53.7%	55.7%
Body mass index (Mean, (SD))	24.1 (3.7)	24.1 (3.8)	24.3 (3.9)	23.9 (3.5)
Country of origin (N, %):				
France	163 (36.1%)	82 (35.8%)	38 (35.2%)	43 (37.4%)
Germany	174 (38.5%)	88 (38.4%)	43 (39.8%)	43 (37.4%)
Spain	115 (25.4%)	59 (25.8%)	27 (25.0%)	29 (25.2%)
ARIA severity (N, %):				
Mild intermittent	54 (12.2%)	29 (12.9%)	10 (9.6%)	15 (13.2%)
Moderate/severe intermittent	147 (33.3%)	81 (36.2%)	38 (36.5%)	28 (24.6%)
Moderate/severe persistent	241 (54.5%)	114 (50.9%)	56 (53.8%)	71 (62.3%)
Number of concomitant conditions reported in the previous 3 months (mean, SD)	0.96 (1.14)	0.96 (1.07)	1.00 (1.25)	0.92 (1.16)
Number of concomitant medications taken in the previous 3 months (mean, SD)	1.60 (0.67)	1.61 (0.67)	1.57 (0.61)	1.60 (0.74)
Number of patients who ever experienced both ocular and nasal symptoms (N, %)	324 (71.7%)	162 (70.7%)	81 (75.0%)	81 (70.4%)

FF: Fluticasone furoate; FP: Fluticasone propionate; MF: Mometasone furoate; SD: Standard Deviation; N: Number of patients.

### Symptom-free days

**Figure 1:**
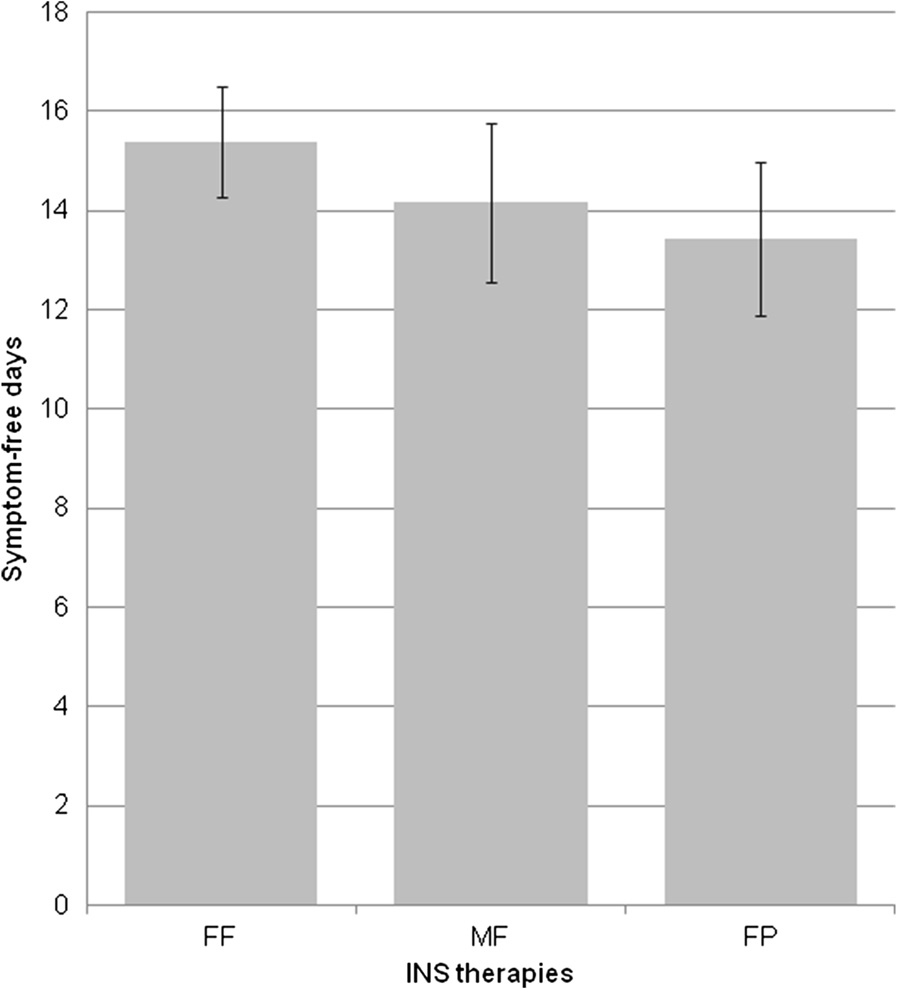
**Number of symptom free days in the past 4 weeks for patients on each INS therapy.** FF: Fluticasone furoate; FP: Fluticasone propionate; MF: Mometasone furoate; INS: intranasal corticosteroids.

The mean number of SFD over the previous 4 weeks reported by the 417 patients that answered this question was 14.58 (±8.42) days. Patients receiving FF were associated with having more SFD (mean 15.45 ±8.29) than patients receiving MF (adjusted mean difference −1.22, 95% Confidence Interval (CI) [−3.16 to 0.72], p=0.434) or FP (adjusted mean difference −1.95, 95% CI [−3.87 to −0.03], p=0.092), although statistical significance was not achieved (Table 2; Figure 1).

**Table 2:** Symptom free days by country and ARIA severity

	**Total**		**FF**		**MF**			**FP**		
	**N**	**Mean (SD)**	**N**	**Mean (SD)**	**N**	**β**	**95% CI**	**N**	**β**	**95% CI**
Symptom-free days	417	14.58 (8.42)	208	15.45 (8.29)	102	−1.22	−3.16 to 0.72	107	−1.95	−3.87 to −0.03
Country sub-groups										
France	152	14.45 (8.74)	75	16.15 (8.43)	36	−3.03	−6.37 to 0.32	41	−2.77	−6.03 to 0.49
Germany	165	16.19 (7.70)	83	16.45 (7.74)	42	−0.81	−3.68 to 2.07	40	−0.59	−3.50 to 2.33
Spain	100	12.12 (8.51)	50	12.74 (8.51)	24	1.48	−2.56 to 5.53	26	−0.98	−4.99 to 3.03
Severity sub-groups										
Mild intermittent	50	18.72 (8.34)	26	20.23 (7.69)	9	−1.77	−9.12 to 5.58	15	−2.30	−8.68 to 4.08
Moderate/severe intermittent	138	15.21 (7.57)	74	15.05 (7.49)	38	1.44	−1.57 to 4.46	26	−1.76	−5.23 to 1.70
Moderate/severe persistent	229	13.30 (8.62)	108	14.56 (8.62)	55	−2.50	−5.29 to 0.28	66	−1.95	−4.58 to 0.69

FF: Fluticasone furoate; FP: Fluticasone propionate; MF: Mometasone furoate; β = adjusted mean difference from FF.

When analyzed by country and ARIA severity, patients on FP reported fewer SFD in all the sub-groups, though none reached statistical significance. Similarly most of the sub-groups of patients receiving MF reported fewer SFD, except Spanish and moderately severe, intermittent sub-groups which were associated with greater SFD. Again, none of these reached statistical significance (Table 2).

### Quality of life

**Figure 2:**
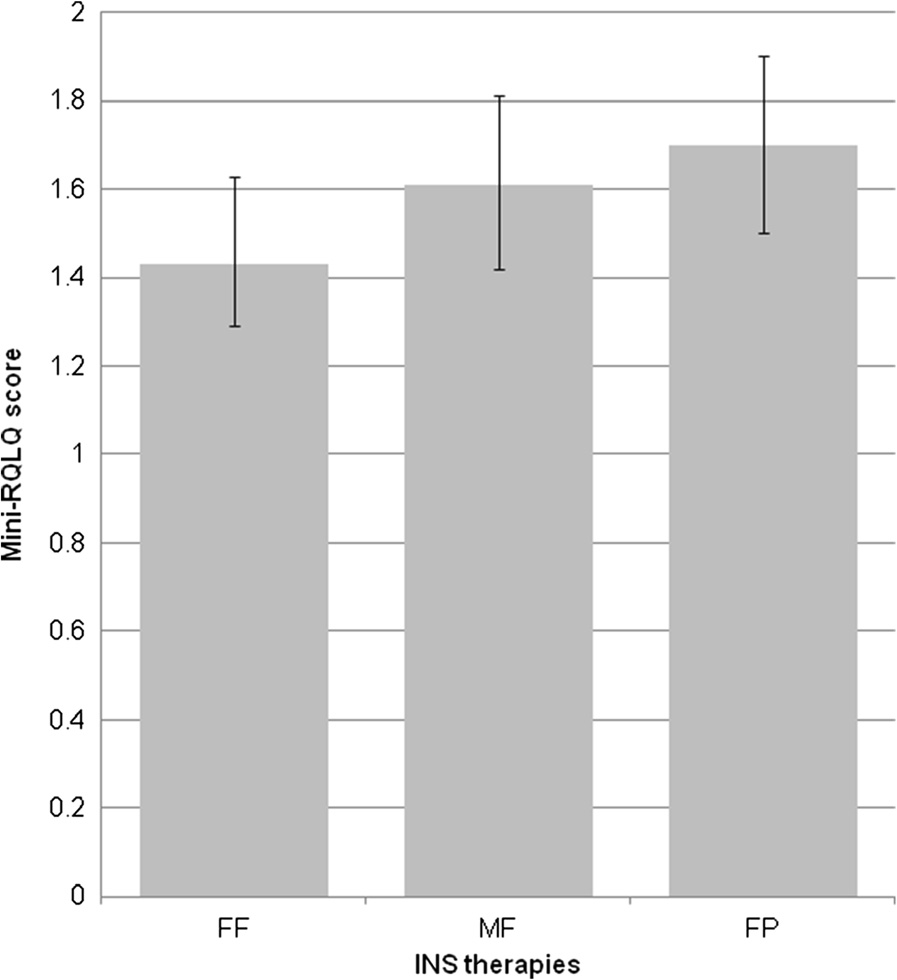
**Mini-RQLQ score for patients on each INS therapy.** Mini-RQLQ: mini-rhinoconjunctivitis Quality of Life Questionnaire; FF: Fluticasone furoate; FP: Fluticasone propionate; MF: Mometasone furoate; INS: intranasal corticosteroids.

The mean mini-RQLQ score reported by the 429 patients that completed the questionnaire was 1.54 (±1.06). Patients receiving FF were associated with having a better quality of life in the previous week (mini-RQLQ score: mean 1.42, ±1.04) than patients receiving MF (adjusted mean difference 0.28, 95% CI [0.03 to 0.52], p=0.052) or FP (adjusted mean difference 0.18, 95% CI [−0.05 to 0.41], p=0.244) Again, none of these results achieved statistical significance (Table 3; Figure 2).

**Table 3:** mini-RQLQ scores by country and ARIA severity

	**Total**		**FF**		**MF**			**FP**		
	**N**	**Mean (SD)**	**N**	**Mean (SD)**	**N**	**β**	**95% CI**	**N**	**β**	**95% CI**
Mini-RQLQ score	429	1.54 (1.06)	217	1.42 (1.04)	98	0.28	0.03 to 0.52	114	0.18	−0.05 to 0.41
Country sub-groups:										
France	155	1.35 (0.98)	79	1.25 (1.02)	33	0.46	0.09 to 0.83*	43	0.13	−0.22 to 0.48
Germany	170	1.57 (0.97)	86	1.48 (0.98)	42	0.12	−0.25 to 0.48	42	0.17	−0.20 to 0.54
Spain	104	1.75 (1.27)	52	1.57 (1.16)	23	0.08	−0.51 to 0.66	29	0.06	−0.50 to 0.63
Severity sub-groups:										
Mild intermittent	52	0.89 (0.80)	28	0.92 (0.89)	9	−0.01	−0.71 to 0.69	15	−0.21	−0.83 to 0.40
Moderate/severe intermittent	143	1.41 (0.98)	78	1.35 (1.01)	37	0.09	−0.28 to 0.46	28	0.24	−0.18 to 0.65
Moderate/severe persistent	234	1.76 (1.09)	111	1.59 (1.06)	52	0.45	0.09 to 0.81*	71	0.22	−0.11 to 0.55

FF: Fluticasone furoate; FP: Fluticasone propionate; MF: Mometasone furoate; β = adjusted mean difference from FF.

*p<0.05 (Bonferroni correction applied).

When analyzed by country and ARIA severity, patients on FF reported a better RQLQ score compared to MF or FP in all sub-groups except in mild intermittent patients. Only the lower RQLQ score for FF compared to MF in French patients (+0.46) and moderately severe persistent patients (+0.45) reached statistical significance (Table 3).

### Other analyses

**Figure 3:**
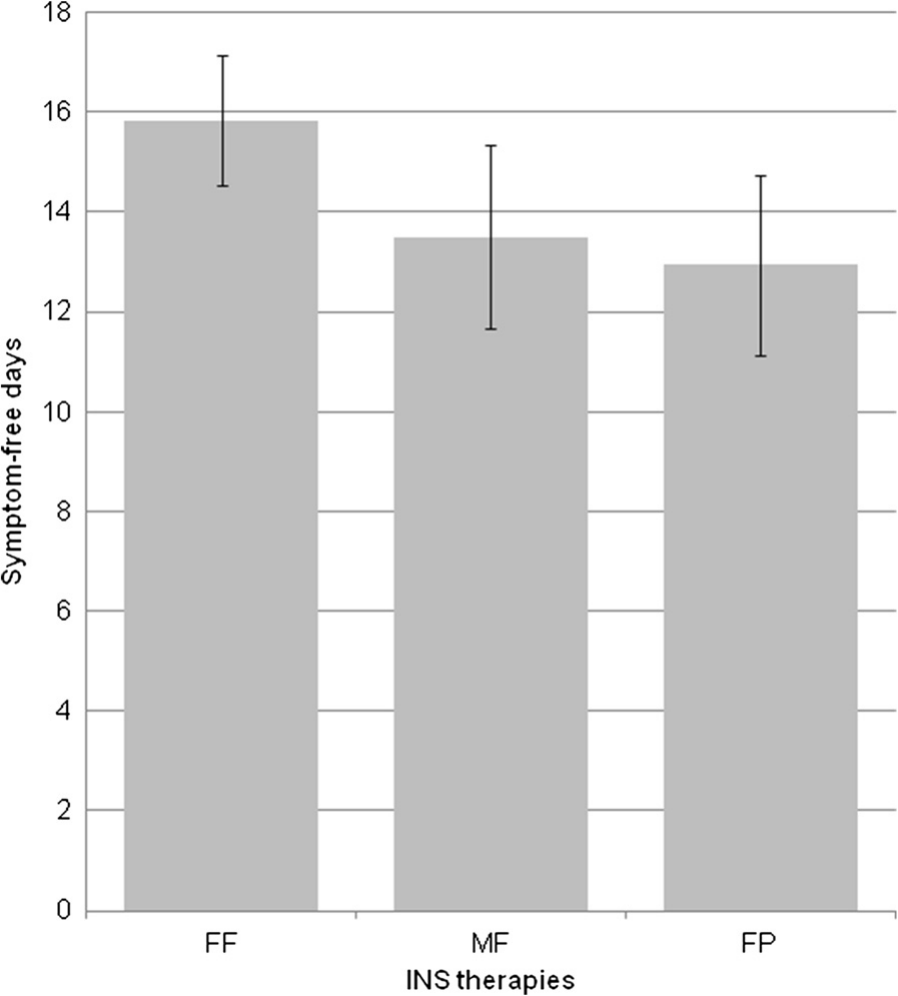
**Symptom free days in patients with both nasal and ocular symptoms.** Number of symptom free days in the past 4 weeks, in patients with a history of both ocular and nasal symptoms on each INS therapy. FF: Fluticasone furoate; FP: Fluticasone propionate; MF: Mometasone furoate; INS: intranasal corticosteroids.

In a post-hoc analysis on those SAR patients (N=324, 71.6%) who had ever experienced both ocular and nasal symptoms, the mean number of SFD was fewer than in the overall study population (14.51 ±8.23). Interestingly those patients receiving FP and MF reported fewer SFD than the overall group of patients on these therapies, while patients receiving FF reported a greater number of SFD than the overall group receiving FF (Figures 1 and 3). Patients in this sub-population who were receiving FF were associated with more SFD than FP (adjusted mean difference −2.34, 95% CI [−4.60 to −0.09], p=0.084) and MF (adjusted mean difference 2.90, 95% CI [−5.13 to −0.67], p=0.022). Similarly, patients in this sub-population, and on each therapy, reported worse RQLQ scores than the overall study population (data not shown). Patients on FF reported better QoL than those patients on FP (adjusted mean difference 0.24, 95% CI [−0.04 to 0.52], p=0.184) or MF (adjusted mean difference 0.28, 95% CI [−0.01 to 0.57], p=0.110), but neither of these were statistically significant.

Overall, patients that completed the PSQI questionnaire reported a mean score of 4.25 (±3.00), which indicates that the majority of responders were not suffering from significant sleep disturbance. In contrast, the mean WPAI scores indicate that allergy was impairing patients’ school or work activities, and mean number of work days lost in the previous 3 months due to allergy was 0.78 (±3.12) (Table 4). Patients reported visiting their primary care physician and specialist physicians in the previous 3 months a mean of 2.06 (±1.72) and 0.96 (±1.37) times, respectively. However, physicians reported a higher visit rate to both the primary care physicians (mean 2.22 ±1.04 visits) and specialists (mean 2.28 ±1.32 times).

Patients on FF reported a better PSQI score than either FP or MF and generally reported less overall impact on work and classroom impairment, except percent work time missed and impairment in the classroom due to allergy. Additionally, patients receiving FF reported fewer work days lost in the previous three months, and fewer patient- or physician-reported visits to PCPs or specialists, than for FP or MF (Table 4). Less OTC drugs were used by patients on FF over the previous 3 months than FP (p<0.05) or MF. However when this was translated into cost spent on OTC products during that time, patients receiving FP only spent €1.50 more, and those receiving MF spent €0.79 less, than patients receiving FF.

**Table 4:** Secondary outcome measures

	**Total**		**FF**		**MF**			**FP**		
	**N**	**Mean (SD)**	**N**	**Mean (SD)**	**N**	**β**	**CI**	**N**	**β**	**CI**
PSQI Overall	370	4.25 (3.00)	183	4.14 (3.14)	87	0.34	−0.37 to 1.06	100	0.08	−0.60 to 0.77
WPAI % work time missed due to allergy	258	1.66 (9.57)	133	2.23 (12.57)	62	−1.31	−4.23 to 1.61	63	−1.10	−4.02 to 1.82
WPAI % impairment while working due to allergy	273	18.06 (16.55)	143	16.99 (16.79)	65	1.33	−3.53 to 6.20	65	3.09	−1.80 to 7.98
WPAI % overall work impairment due to allergy	255	18.93 (18.27)	131	17.88 (19.29)	62	1.67	−3.86 to 7.21	62	2.43	−3.13 to 7.98
WPAI % class time missed due to allergy	55	1.97 (5.99)	27	1.36 (4.09)	16	1.30	−2.80 to 5.40	12	−1.14	−5.98 to 3.70
WPAI % impairment in the classroom due to allergy	61	17.05 (17.16)	31	16.77 (18.51)	16	−5.33	−15.89 to 5.24	14	−0.81	−12.91 to 11.28
WPAI % overall classroom impairment due to allergy	55	17.37 (17.73)	27	16.44 (17.96)	16	−2.96	−14.69 to 8.76	12	1.02	−12.83 to 14.87
WPAI % activity impairment due to allergy	425	20.16 (16.86)	215	18.42 (16.95)	100	3.20	−0.77 to 7.16	110	3.24	−0.62 to 7.09
Number of work days lost in the previous 3 months due to AR	297	0.78 (3.12)	151	0.59 (1.75)	70	0.55	−0.36 to 1.45	76	0.37	−0.51 to 1.25
Number of PCP visits, as reported by patient, in the previous 3 months	436	2.06 (1.72)	220	1.95 (1.36)	102	0.13	−0.27 to 0.53	114	0.23	−0.16 to 0.62
Number of specialist visits as reported by patient, in the previous 3 months	437	0.96 (1.37)	220	0.88 (1.24)	103	0.15	−0.16 to 0.47	114	0.09	−0.21 to 0.40
Number of PCP visits, as reported by physician, in the previous 3 months	283	2.22 (1.04)	144	2.19 (0.98)	66	0.09	−0.21 to 0.38	73	−0.12	−0.40 to 0.17
Number of specialist visits as reported by physician, in the previous 3 months	153	2.28 (1.32)	76	2.16 (1.18)	36	0.37	−0.13 to 0.87	41	0.17	−0.30 to 0.65
Number of non-prescribed (OTC) drugs over the previous 4 weeks	437	0.24 (0.53)	220	0.19 (0.45)	103	0.05	−0.06 to 0.19	114	0.12	0.01 to 0.23*

FF: Fluticasone furoate; FP: Fluticasone propionate; MF: Mometasone furoate; β = adjusted mean difference from FF.

*p<0.05 (Bonferroni correction applied).

## Discussion

Our study demonstrates the impact of allergic rhinitis on symptoms and quality of life in SAR patients who are being treated with INS. The mean number of symptom free days in the overall population was higher than has been previously reported [2,26] for AR patients, indicating that these three treatments provide additional relief from AR symptoms for patients. Similarly the mean RQLQ scores were generally less than has been reported previously in AR patients, particularly those with moderate/severe disease [2,23]. However, at the height of the pollen season, patients still suffer from symptoms nearly 1 in every 2 days, with moderate/severe patients suffering more than mild patients.

Not achieving statistical significance for the majority of comparisons between FF and FP or MF obviously weakens any conclusions that can be drawn as to superiority of any one INS product, and we therefore apply caution in the interpretation of the results presented. FF was associated with more SFD and a better RQLQ than FP or MF. A difference of nearly two days of SFD in a four week period may be considered clinically relevant, as this equates to two additional days the patient is able to live an active life. However, the differences in RQLQ score are unlikely to represent a clinically relevant improvement in QoL as the minimally important difference for the mini-RQLQ is 0.70 [22].

In addition, the inherent variability that occurs within real world research (compared to the artificial environment of randomized controlled trials) can make results from these types of studies appear weak. However, corroborating trends of greater SFD and better QoL of those patients receiving FF across country and ARIA severity sub-groups provide tentative evidence that differences may exist.

Patients who reported ever having suffered from both ocular and nasal symptoms were considered a population of interest in the light of increasing importance of managing both these conditions in AR patients. As reported elsewhere, these patients generally report worse symptom burden and quality of life to the general AR patients [9]. In our population, we observed a drop in the mean number of reported symptom free days for those patients on MF and FP, but not for FF. Similarly, the mean RQLQ scores were higher in these patients compared to the overall study population, confirming that patients who suffer both nasal and ocular symptoms are more burdened by their disease. Within this group, FF continues to show a higher number of SFD and QoL compared to FP and MF.

No significant differences could be discerned between patients receiving FF and those receiving either FP or MF from the secondary outcomes of sleep, work/classroom productivity questionnaires or visits to healthcare professionals. There is likely to be significant variability in these results as patients can have disturbed sleep, not go to work or visit a healthcare professional for many different reasons which may be difficult to put down solely to their allergic rhinitis. However, it is interesting to note that of the 12 domains assessed, the patients in the FF arm reported less impact of AR on their lives than FP and MF in all but 4 (FP) and 3 (MF) domains.

To date there have been very few studies directly comparing INS regimens in patients with SAR [27], and these have mostly compared FF with FP. A Japanese study comparing 2 weeks of treatment with FF and FP in AR patients showed FF once daily was non-inferior to FP twice daily in change in total nasal symptom scores. There were also similar improvements in rhinoscopy findings, activity of daily life interference, and patient-rated evaluation of therapy in the FF and FP groups [28]. Similarly, a US patient preference study showed both FF and FP significantly improved symptoms in adult patients with SAR. Most patients preferred the sensory attributes of FF to those of FP after one week of treatment [29]. Finally, in a large US study on concomitant medications, FF was shown to reduce the need for concomitant AR medications compared with other leading branded INS therapies, including MF and FP [30]. This last study supports some of the data we present here, where we found patients receiving FF required significantly fewer non-prescription (OTC) medications over the previous four weeks than patients receiving FP.

No direct comparison of the efficacy of INSs on ocular symptoms exists, but a recent review of available data highlighted that the data supporting FF consistently showed positive effects, while conflicting, inconsistent or negative effects were observed with the other INSs examined [14]. Further studies directly comparing efficacy on nasal and ocular symptoms, improvement in QoL and patient preferences of INS regimens and even anti-histamines, in subjects with AR, are needed.

### Limitations

A number of limitations in this study design exist. The diagnosis of SAR was based on the physicians’ diagnosis rather than a formalized definition of the condition, which may have allowed patients who would not have met a strict definition of SAR of a clinical study to participate. Additionally, a patient selection bias may exist in the study, as the study population represents a convenience sample. It is unlikely to be representative of the overall population of patients with SAR, although it should be representative of the consulting population. While studies such as ours focus on a self-selecting sub-set of SAR patients, it is probable that those patients who do consult with their PCP are those most bothered by their symptoms, and therefore represent a population most in need of effective treatment.

This was a real world design which was reliant on patients having been sufficiently adherent to their treatment and their recollection of their health over a period of time. This can introduce recall bias although there was no reason to assume this would affect one study population more than another.

As the study was cross-sectional it is not possible to derive a causal relationship between drug and effect, nor do we know whether patients prescribed FF were originally more or less burdened by their disease than their counterparts who were prescribed FP or MF. Neither did we collect information on time since diagnosis, or time on treatment, which similarly may have impacted the study results. However, we are able to infer association whilst taking into consideration confounding variables to strengthen conclusions drawn from the associations generated. Additionally, SAR is not considered to be a progressive disease so current severity evaluation usually acts as a proxy for severity of disease. This study design also does not allow us to assess any change over time, or investigate whether these results hold true during periods of high or low pollen counts. A baseline severity may have allowed comparisons between the treatments to be made between the arms with more confidence, however a retrospective baseline would have been subjective and open to interpretation, so it was not included in the study.

The sample size for the study was based on the practicalities of conducting the study, and not on a formal power calculation or adjusting for multiplicity. In retrospect, the sample size may not have been sufficiently large to allow for statistical investigation of the effects of the different therapies over a large number of variables. Given that the main focus was on significant results, confidence that these results will generalise to independent data is weaker than if analysis involved a single comparison only. However, the totality of our results provides directional evidence that FF performs better than either FP or MF across a range of criteria in this population, which is consistent with the randomised controlled trial results.

## Conclusions

At the height of the allergy season, patients being treated for SAR still suffer symptoms and report an impact on their QoL. SAR patients who had ever suffered from ocular and nasal symptoms, reported fewer SFD and a poorer QoL than the general SAR population. Directional evidence is presented to show FF is associated with more SFD and a better QoL than both FP and MF. However, the absence of statistical significance weakens the strength of any interpretation other than no key significant differences were observed between FF, FP and MF.

## Abbreviations

AR: Allergic rhinitis; ARIA: Allergic rhinitis and its impact on asthma; BMI: Body mass index; CI: Confidence interval; COPD: Chronic obstructive pulmonary disease; EphMRA: European pharmaceutical market research association; FF: Fluticasone furoate; FP: Fluticasone propionate; HCP: Healthcare professional; INS: Intranasal corticosteroids; MF: Mometasone furoate; OLS: Ordinary least square; OTC: Over-the-counter; PCP: Primary care physician; PSQI: Pittsburgh sleep quality index; QoL: Quality of life; RQLQ: Mini-rhinoconjunctivitis quality of life questionnaire; SAR: Seasonal allergic rhinitis; SD: Standard deviation; SFD: Symptom-free days; US: United States; WPAI+CIQ AS: Work productivity and activity impairment: allergy specific questionnaire plus classroom impairment questions: allergy specific.

## Competing interests

MS and JP are employees of Adelphi Real World, who conducted the research, and received no financial compensation from GlaxoSmithKline.

PD is a consultant and/or a speaker for Stallergenes, ALK, Circassia, Allergopharma, Chiesi, Schering-Plough-MSD, AstraZeneca, Pierre Fabre Médicaments, Ménarini and GlaxoSmithKline.

HM is an employee of, and holds shares in, GlaxoSmithKline, the manufacturer of both fluticasone furoate and fluticasone propionate nasal sprays.

## Authors’ contributions

MS and JP designed and supervised collection of the data, collaborated closely with the statistician in data review and critically reviewed the content of the manuscript. PD provided medical input into the study and data interpretation. HM provided scientific input into the data interpretation and drafted the manuscript. All authors critically reviewed and approved the final manuscript.

## Authors’ information

Pascal Demoly, MD, PhD is Professor of Pulmonology at the University of Montpellier, France and Head of the Department at the University Hospital of Montpellier. In the last years his research interest has focused mainly on drug allergy, allergic rhinitis and asthma diagnosis and treatment. He has published more than 500 articles including more than 220 in international peer-reviewed journals and is a WHO expert for allergy (International Consensus on Specific Immunotherapy, Allergic Rhinitis and its Impact on Asthma). He is the Vice-President for Education & Specialties of EAACI.
